# Abstracts

**DOI:** 10.1155/S1463924684000249

**Published:** 1984


Journal of Automatic Chemistry, Volume 6, Number 3 (July–September 1984), pages 119–121

### Abstracts

### Page 122

**The evaluation kit for clinical chemistry: a practical guide for the evaluation of methods, instruments and reagent kits**
G. H. White and C. G. Fraser

This article does not have an abstract so to help readers we include French and German versions of its first section 'Why evaluate'?

**Une méthode de jugement pour la chimie clinique: un guide pratique pour le jugement de méthodes, d'instruments et de réactifs**
G. H. White and C. G. Fraser

Le premier rôle du chimiste clinique est de procurer des services de laboratoire diagnostique qui permettent les meilleurs soins possibles selon la technologie accessible et les facteurs économiques locaux. Cependant, un service acceptable est assuré seulement si des standards élevés de performance analytique sont réalisés et maintenus. Pour contrôler la performance analytique, la plupart des laboratoires utilisent des programmes internes ou externes pour contrôle de qualité. La stratégie de ces méthodes est de comparer la performance actuelle avec la performance initiale de la méthode et ne pas de procurer des données sur la méthode elle-même c.-à-d. de contrôler si elle remplie les standards de performance analytiques et cliniques requis.

Donc, avant d'introduire une méthode dans les services de routine dans n'importe quel laboratoire, même le plus petit, il est nécessaire de qualifier objectivement la méthode elle-même, pas seulement ses caractéristiques analytiques et sa capabilité de remplir une nécessité clinique, mais également son influence sur le laboratoire, le personnel et le budget. Afin d'obtenir les données qui permettent de définir complètement une méthode, il est nécessaire d'éffectuer une évaluation complète de celle-ci. Il est préfèrable de faire une telle évaluation avant que la méthode (que ce soit un instrument ou un set de réactifs) est achetée, mais il est également nécessaire d'évaluer des méthodes déja achetées, même depuis un certain temps, de la même façon précise. Une fois que l'évaluation est faite, la méthode est complètement caractérisée. Ces données sont alors retenues dans le laboratoire; maintenant les programmes de contrôle de qualité peuvent être instaurées et utilisées pour assurer que la performance analytique bien définie et acceptée de cette méthode soit maintenue.

Le processus complet d'évaluation devrait être une progression logique à travers les différentes étapes de l'étude avant l'achat, la familiarisation, l'évaluation et le jugement objectif de l'acceptabilité. Aucune des ces étapes devrait être omise alors qu'une méthode est introduite dans le service de routine.

Beaucoup de protocoles d'évaluation ont été publiés dans la littérature de la chimie clinique. La plupart sont très bien étudiés et bien décrits. Ils ont, cependant, tendance d'être écrits pour l'expert, et, par conséquence, sont souvent assez théoriques. Un guide de travail compact pour le chimiste clinique pratique a manqué jusqu'à ce jour. Cette publication procure un tel guide pratique comme une carte pour le chauffeur qui n'a jamais utilisé une certaine route.

Cette méthode d'évaluation est facile à employer. La section II contient les expressions utilisées dans la théorie et pratique d'évaluation; elle peut être omise par ceux familiers avec la théorie. Les sections III à IX procurent un guide détaillé commençant par l'itinéraire et ensuite progressant de façon logique à travers les différentes étapes du processus d'évaluation.

Ce matériel se base sur l'expérience pratique, sur la théorie, sur les publications préalables et sur des recommandations nationales et internationales actuelles. Les principales sources d'information sont mentionnées dans la bibliographie (Section X).

**Das Auswertungspaket für die klinische Chemie: Eine praktische Anleitung für die Beurteilung von Methoden, Instrumenten und Reagentienpaketen**
G. H. White and C. G. Fraser

Die primäre Rolle des klinischen Chemikers besteht darin, sicherzustellen, dass diagnostische Labordienstleistungen zur Verfügung stehen, die eine optimale Patientenbetreuung ermöglichen und die so gut sind als es die verfügbare Technik und die lokalen ökonomischen Faktoren gestatten. Eine akzeptable Dienstleistung liegt aber nur dann vor, wenn ein hoher Stand der analytischen Leistung erreicht und beibehalten wird. Um die analytische Leistung zu überwachen, benützen die meisten Laboratorien umfassende interne und externe Qualitätssicherungsprogramme. Es sollte aber nicht vergessen werden, dass die Strategie solcher Programme aus dem Vergleich der heutigen mit der gestrigen Leistung besteht und nicht Daten dazu liefert, ob die Methode als solche überhaupt den notwendigen analytischen und klinischen Leistungsanforderungen zu genügen vermag.

Bevor eine Methode somit in den Routinebetrieb irgendeines Labors, auch des kleinsten, gebracht wird, ist es notwendig die Methode objektiv zu beurteilen und zwar nicht nur in Bezug auf die analytischen Merkmale und der Fähigkeit klinische Anforderungen zu erfüllen, sondern ebenso in Bezug auf den Einfluss auf Laborpersonal und Budget. Es muss eine saubere und vollständige Evaluation durchgeführt werden, um die Daten für eine voll definierte Methode zu erarbeiten. Es ist empfehlenswert, vor dem Kauf einer Methode (ob Instrument oder Reagenspaket) eine solche Auswertung durchzuführen; eine solche genaue Evaluation sollte aber ebenfalls bei bereits gekauften Methoden zur Anwendung gelangen, selbst wenn sie seit einiger Zeit in Gebrauch sind. Sobald die Evaluation vorliegt, ist die Methode voll charakterisiert. Die erhaltenen Daten können dann im Labor aufbewahrt werden. Nur so können Qualitätsüberprüfungs- und Qualitätsicherungsprogramme erstellt und benützt werden, die garantieren, dass eine definierte und akzeptable analytische Leistung der Methode aufrecht erhalten bleibt.

Der gesamte Evaluationsprozess sollte eine logische Folge der Schritte Beurteilung vor Kauf oder Service, Familiarisierung, Evaluation und objektive Beurteilung der Akzeptierbarkeit sein. Bevor eine mögliche Methode zur Routine erhoben wird, sollte keine dieser Stufen ausgelassen werden.

Viele Evaluationsprotokolle wurden in der Literatur der klinischen Chemie publiziert. Die meisten davon sind gut durchdacht und sorgfältig geschrieben. Sie neigen aber dazu, für den Experten geschrieben worden zu sein, und sind demzufolge etwas theoretisch. Eine kurze Arbeitsanleitung für den klinischen Laien hat aber bisher gefehlt. Diese Publikation liefert nun eine solche praktische Anleitung.

Das Auswertepaket ist einfach zu gebrauchen. Abschnitt II enthält die Ausdrücke und Redewendungen welche in der Theorie und Praxis der Evaluation gebraucht werden; dies kann von jenen, die mit dem Gebiet bereits vertraut sind, übersprungen werden. Die Abschnitte III bis IX liefern eine detaillierte Wegbeschreibung, welche mit der Route beginnt und danach auf logische Art und Weise durch die Schritte des Evaluationsverfahrens führt.

Das Material basiert auf praktischen Erfahrungen, auf der Theorie, auf bereits publizierten Arbeiten und auf den gegenwärtigen nationalen und internationalen Empfehlungen. Grössere Informationsquellen sind in der Bibliographie angeführt.

---

**Page 142**

**Automation in monitoring gases and particulates in the pulp and paper industry**
T. L. C. de Souza

The Canadian pulp and paper industry is required to monitor the pollution it generates of water, air and land. This paper deals with what this industry has done, and is trying to do, in automating the monitoring of pollutant gases and particulate matter. The use of continuous or automated instruments usually cuts back on manpower requirements, while rigid maintenance becomes necessary because of the complexity of operating some of these sophisticated pieces of equipment.

**Automation du contrôle des gaz et des particules dans l'industrie du papier**
T. L. C. de Souza

L'industrie canadienne du papier doit, de plus en plus, contrôler la pollution de l'eau, de l'air et de la terre qu'elle génère. Cette publication présente ce que cette industrie a fait et ce qu'elle essaie de faire pour automatiser le contrôle des polluants gazeux et solides.

L'utilisation d'instruments en continu ou automatiques en général réduit le nombre de personnes requises, tandis qu'une maintenance sévère devient nécessaire à cause de la complexité d'opérations de certains de ces systèmes sophistiqués.

**Automation für die Überwachung von Gasen und Festteilchen in der Holz- und Papierindustrie**
T. L. C. de Souza

Die kanadische Holz- und Papierindustrie ist genötigt, die von ihr verursachte Umweltverschmutzung zu Wasser, Luft und Land zu überwachen. Diese Arbeit befasst sich mit dem, was die Industrie in der Automation der Ueberwachung von verunreinigenden Gasen und Festteilchen unternommen hat und zu unternehmen versucht. Die Verwendung von kontinuierlich arbeitenden oder automatisierten Geräten reduziert im allgemeinen den Personalbedarf, verlangt aber auf der anderen Seite wegen der Komplexität der Bedienung einiger dieser ausgeklügelten Geräte einen strikten Unterhalt.

---

**Page 148**

**A microprocessor data-capture and database package for the IL508 analyser**
B. Clark *et al.*

The use of an Apple II microcomputer as an on-line data-capture device for an IL508 analyser is described. The package is equipped with a true data-base, so 650 records may be on-line at any given time for editing or printing with a random access time of several seconds. Quality-control data is also held in the file and may conveniently be accessed for processing. The package provides for flexible output formats to the computer screen, and for printing on plain paper or sticky labels, so that each medium is optimally catered for. Facilities are also provided to send relevant data to a PDP 11/34 computer as required.

**Un système à microprocesseurs pour réception et traitement de données pour l'analyseur IL508**

B. Clark *et al.*

L'utilisation d'un miniordinateur Apple II comme instrument pour réception de données en ligne pour un analyseur IL508 est décrite. Le système est équipé d'un vrai fichier de base, tel que 650 enregistrements sont accessibles à tout moment pour traitement ultérieur ou impression avec un temps d'accès de quelques secondes. Les données de contrôle de qualité sont également mémorisées et sont facilement accessibles pour traitement ultérieur. Le système prévoit différents formats flexibles pour las présentation sur l'écran et pour l'impression sur papier ou étiquettes auto-collantes, avec présentation optimale pour chaque application. Le transfert de données vers un PDP 11/34 est également prévu.

**Mikroprozessor-Datenerfassung und-Datenbank Software-Paket für den IL508 Analysator**
B. Clark *et al.*

Die Arbeit beschreibt die Verwendung eines Apple II Mikrocomputers für die on-line Erfassung der Daten eines IL508 Analysators. Das Paket ist mit einer echten Datenbank ausgerüstet, mit der jederzeit 650 Records on-line mit einer mittleren Zugriffszeit von einigen Sekunden für Editieren und Ausdruck zur Verfügung stehen. Qualitätssicherungsdaten sind in der Datenbank ebenfalls enthalten und sind für eine Verarbeitung leicht zugreifbar. Das Paket ist mit einer flexiblen Formatierung für die Ausgabe auf dem Bildschirm oder den Ausdruck auf Papier oder Klebeetiketten versehen, sodass jedes dieser Medien optimal berücksichtigt ist. Es wurden auch Vorkehrungen getroffen, dass relevante Daten nach Bedarf an einen PDP 11/34 übermittelt werden können.

---

**Page 152**

**Automated enzymatic determination of plasma free fatty acids by centrifugal analysis**
D. P. Knox and D. G. Jones

An enzymatic micromethod for the rapid routine estimation of free fatty acids (FFA) in plasma or serum using an I L Multistat III microcentrifugal analyser is described. Apart from the advantages of speed (18 tests/5 min) and convenience, the use of bichromatic within-sample blanking and small reagent volumes (240 $\mu$l/test) makes the method extremely cost-effective compared with existing manual methods.

Reaction conditions were optimized to give a linear reponse for plasma FFA concentrations between 0·1 and 2·0 mmol/litre. Within-run and between-run coefficients of variation were consistently less than 5% and there was a good correlation with a manual colorimetric method. Plasma, with oxalate/fluoride or EDTA as anticoagulant, and serum gave similar FFA values, but the presence of heparin caused overestimation by about 10%. FFA in plasma or serum were stable for at least two weeks at $-20$°C, and for at least two days at 4°C, but were unstable at room temperature.

*French and German abstracts will be published in Vol. 6, No. 4.*

---

**Page 155**

**A computer program for intra-laboratory quality control**
C. Zuppi *et al.*

An intra-laboratory quality-control system is described; mathematical and graphical procedures have been automated by means of a minicomputer. The system has proved itself to be both effective and timely. A simple and unequivocal interpretation of control results has been obtained by using an effective statistical parameter: the reliability index.

### Un programme d'ordinateur pour le contrôle de qualité au laboratoire

C. Zuppi *et al.*

Un système de contrôle de qualité au laboratoire est décrit. Des processus mathématiques et graphiques ont été automatisés à l'aide d'un miniordinateur. Le système a prouvé d'être efficace et actuel. Une interprétation simple et non équivoque des résultats de contrôle a été obtenue en utilisant un paramètre statistique effectif: l'indice de sûreté.

### Ein Rechnerprogramm für die Qualitätskontrolle im Labor

C. Zuppi *et al.*

Die Arbeit umfasst die Beschreibung eines Qualitätskontroll-systems für das Labor. Mathematische und graphische Prozeduren wurden mit Hilfe eines Minicomputers automatisiert. Das System erwies sich als effizient und zeitgerecht. Eine einfache und klare Interpretation der Kontrollresultate wurde erhalten durch die Verwendung eines wirksamen statistischen Parameters: der Zuverlässigkeits-Index.

---

### Page 158

**Experiences with a system for signal- and data-processing, together with on-line variance reduction, in continuous-flow analysis**

R. Maasen *et al.*

The authors describe a microcomputer read-out interface for a continuous-flow analyser; it is an adapted version of a previously described configuration. Two new features have been added to the program. A subroutine which corrects for base-line drift means a higher reliability. The main new feature is a subroutine that reduces between-run variance by, on average, 30–40%. A digital filtering technique estimates the varying process fluctuations by processing the results of two control sera present in every sample batch to be analysed. The original flexibility requirements, no restrictions on the run length and the absence of any restricted position in the run, are preserved.

**Expériences avec un système pour le traitement de signaux et de données en même temps qu'avec une réduction de la variance online dans un système d'analyse à flux continu**

R. Maasen *et al.*

Les auteurs décrivent une interface pour microordinateurs pour analyseurs à flux continu qui est une version adaptée d'une configuration déjà décrite. Deux nouvelles possibilités ont été ajoutées au programme. Une subroutine qui corrige la dérive de la ligne de base donne une meilleure sûreté. La principale nouvelle possibilité consiste d'une subroutine qui réduit la variance entre séries d'environ 30 à 40%. Une technique de filtrage digital estime les fluctuations variantes du processus en traitant les résultats de 2 sérums de contrôle présents dans chaque série d'echantillons qui doit être analysée. La flexibilité du système original qui ne pose aucune restriction à la longueur de la série et l'absence de restrictions sur la position dans la série sont préservées.

**Erfahrungen mit einem System für die Signal- und Datenverarbeitung zusammen mit einer on-line Varianz-Reduktion in der kontinuierlichen Durchflussanalyse**

R. Maasen *et al.*

Die Autoren beschreiben ein Datenerfassungs-Interface mit Mikrocomputer für einen kontinuierlichen Durchflussanalysator, welches eine neu angepasste Version einer früher beschriebenen Konfiguration ist. Dem Programm wurden zwei neue Eigenschaften beigefügt. Ein Unterprogramm, das den Basislinien-Drift korrigiert, ergibt eine höhere Zuverlässigkeit. Die neue Haupteigenschaft besteht aus einem Unterprogramm, das die Varianz zwischen den Läufen um durchschnittlich 30–40% reduziert. Mit einer digitalen Filtertechnik werden die variierenden Prozessschwankungen geschätzt, indem die Resultate von zwei Kontrollseren, die in jeder zu analysierenden Probenserie vorhanden sind, verarbeitet werden. Die ursprünglichen Flexibilitätserfordernisse, keine Einschränkung der Serielänge und das Fehlen eingeschränkter Positionen in der Serie, bleiben erhalten.

---

### Page 161

**Erythrocyte transketolase activity measured on the Gemstar discrete analyser**

J. E. Buttery *et al.*

The kinetic method of Smeets *et al.* [1] for erythrocyte transketolase (TK) was adapted for the Gemstar, a compact table-top discrete analyser. Results by both methods were compared using neat and thiamine activated haemolysate samples.

The correlation (r) for the TK assay was 0·98 and the least squares regression according to Deming was $y=0{\cdot}85x+0{\cdot}144$, while for the thiamine activated TK assay, $r=0{\cdot}98$ and $y=0{\cdot}90x+0{\cdot}048$. The within-day precision was good; the coefficient of variations were 4·9% and 3·0% at two activity levels. The reference range for erythrocyte TK activity is 0·6 to 1·3 U/g Hb and the thiamine activation is 2 to 25%.

**Activité de la transkétolase d'érythrocites mesurée sur l'analyseur discret Gemstar**

J. E. Buttery *et al.*

La méthode cinétique de Smeets *et al.* [1] pour la transkétolase d'érythrocytes (TK) a été adaptée pour le Gemstar, un analyseur discret compact se plaçant sur la table. Les résultats des deux méthodes ont été comparés utilisant des échantillons d'hémolysate pure et activés par thiamine.

La corrélation (r) pour l'analyse TK était 0·98 et la regression aux moindres carrés suivant Deming était $y=0{\cdot}85x+0{\cdot}144$, pendant que pour l'analyse TK activée par thiamine $r=0{\cdot}98$ et $y=0{\cdot}90x+0{\cdot}048$. La précision pendant la journée était bonne, le coéfficient de variation était 4·9% et 3·0% à deux niveaux d'activité différents. La gamme de référence pour l'activité d'érythrocites TK est de 0·6 à 1·3 U/g Hb et l'activation de thiamine est de 2 à 25%.

**Bestimmung der Erythrocyten Transketolase-Aktivität mit dem diskontinuierlichen GEMSTAR Analysengerät**

J. E. Buttery *et al.*

Die kinetische Methode von Smeets *et al.* [1] für die Bestimmung der Erythrocyten Transketolase (TK) wurde auf das GEMSTAR Gerät, einem diskontinuierlichen, kompakten Tischgerät, angepasst. Die Resultate der Analysen von reinen und mit Thiamin aktivierten Haemolysat-Proben wurden miteinander verglichen.

Die Korrelation (r) für den TK-Test betrug 0·98 und die Regression der kleinsten Quadrate nach Deming $y=0{\cdot}85x+0{\cdot}144$, während sich für den Thiamin aktivierten TK-Test $r=0{\cdot}98$ und $y=0{\cdot}90x+0{\cdot}048$ ergab. Die Präzision innerhalb eines Tages war gut; die Variationskoeffizienten betrugen 4·9% und 3·0% für zwei Aktivitätsniveaus. Der Referenzbereich für Erythrocyten TK-Aktivität beträgt 0·6–1·3 U/g Hb und die Thiamin Aktivierung 2–25%.

